# Effect of dexmedetomidine infusion for intravenous patient-controlled analgesia on the quality of recovery after laparotomy surgery

**DOI:** 10.18632/oncotarget.22232

**Published:** 2017-11-01

**Authors:** Juan Xin, Yabing Zhang, Leng Zhou, Fei Liu, Xiaoshuang Zhou, Bin Liu, Qian Li

**Affiliations:** ^1^ Department of Anesthesiology, West China Hospital of Sichuan University, Chengdu, Sichuan, China

**Keywords:** dexmedetomidine, patient-controlled analgesia, QoR-15

## Abstract

**Background:**

The Quality of Recovery-15 (QoR-15) is a patient-centered questionnaire to evaluate the recovery after surgery and anesthesia. Dexmedetomidine has sedative, analgesic, antiinflammatory and inhibitory sympathetic effects, which may contribute to early recovery. We hypothesized dexmedetomidine added to intravenous patient-controlled analgesia (PCA) could enhance the quality of recovery (QoR) in patients undergoing laparotomy surgery.

**Methods:**

In this randomized, double-blind, controlled study, 100 patients undergoing laparotomy surgery were randomly allocated into two groups: Dexmedetomidine (group D) and control (group S). Patients in the group D (n = 46) received dexmedetomidine 0.04 ug/(kg·h) plus sufentanil 0.02 ug/(kg·h) for 48 h after laparotomy surgery, and in the group S (n = 47) received sufentanil 0.04 ug/(kg·h) only. The QoR-15 scores, postoperative pain, rescue analgesia, recovery of gastrointestinal function, patient satisfaction and adverse effects were recorded.

**Results:**

The QoR-15 scores were significantly higher in the group D than in the group S on postoperative day (POD) 1, 2, 3 and 5 (P < 0.05). The visual analog scale (VAS) scores were significantly lower in the group D compared with group S within 48 h after surgery (P < 0.05). The pressing times of analgesic pump and rescue tramadol were not significantly different between the two groups (P > 0.05). The incidence of nausea was significantly lower in the Group D. No hypotension, bradycardia, or respiratory depression was observed.

**Conclusions:**

The addition of dexmedetomidine to PCA enhanced patient-centered recovery, reduced pain and adverse effect, and improved patient satisfaction after laparotomy surgery.

## INTRODUCTION

Laparotomy, one of the most common surgical procedures, is widely used in clinical practice, which is the preferred choice for abdominal sepsis and abdominal compartment syndrome [1, 2]. Laparotomy would cause great damage, associated with a high incidence of postoperative pain, increase the incidence of complications and thus delay the process of postoperative recovery [3, 4]. Promoting early recovery has important clinical significance, which is one of the most important medical tasks.

Dexmedetomidine, a highly selective α2-adrenoceptor activation, is a sedative, analgesic, pathologic anxiety relieving, and anti-inflammatory drug, without respiratory depression and opioid-sparing effect [5-7]. Dexmedetomidine is effective, alone or in combination with other analgesics, in reducing postoperative pain [8-10]. Furthermore, dexmedetomidine has been shown to decrease nausea and vomiting, improve mood and speed up patient recovery in a variety of medical and surgical patients [8, 9].

It is becoming increasingly important to measure the quality of recovery (QoR) from the perspective of the patient. Most recent studies focused on recovery time, pain, or other adverse reactions, however, these are not enough to reflect the recovery of the patient from anesthesia and surgery. The QoR-15, which is a patient-centered QoR measure, can effectively evaluate the quality of postoperative rehabilitation [11, 12]. We hypothesized that intravenous patient-controlled analgesia (PCA) with dexmedetomidine would beneficially affect patient-centered QoR and that several early clinical recovery variables during hospitalization, such as pain, nausea, and patient satisfaction.

## RESULTS

### Demographic data and surgery/anesthesia-related information

**Figure 1:**
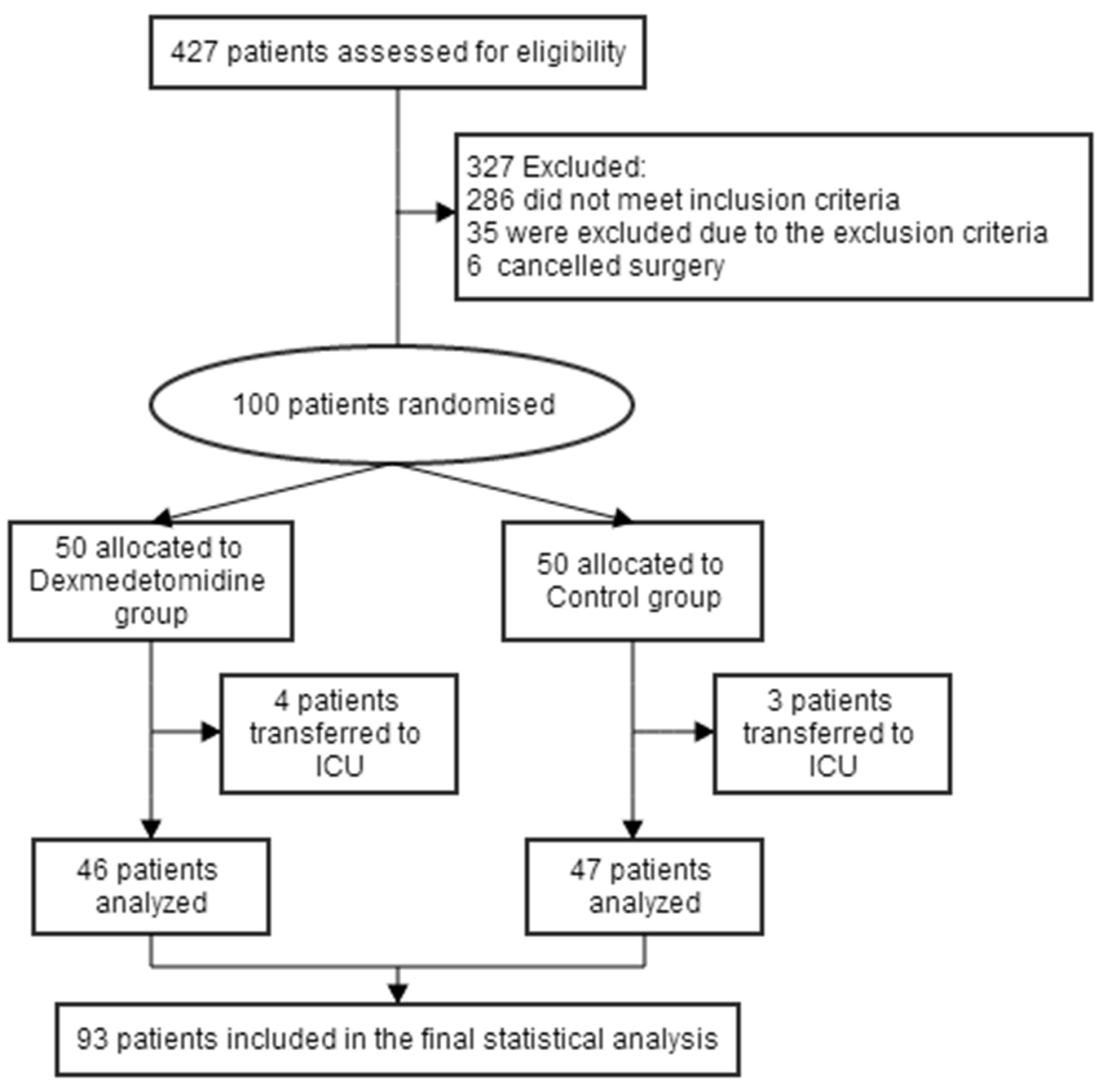
Flow diagram of the study

Between December 2016 and May 2017, of 427 patients screened, a total of 100 patients were enrolled. With 7 patients excluded, 93 (93.0%) patients were included in the statistical analysis: 46 patients in group D and 47 patients in group S (Figure 1). There were no significant difference between the two groups in patient characteristics and intraoperative variables were similar (P > 0.05; Table 1).

**Table 1:** Patient characteristics and intraoperative data

	Group S (n=47)	Group D (n=46)	Difference (95% CI)	P Value
Sex, male:female	27 (57.4%):20 (42.6%)	24 (52.2%):22 (47.8%)		0.609
Age, yr	54.6 ± 14.6	51.2 ± 15.1	3.3 (-2.8 to 9.4)	0.290
Height, cm	167.4 ± 6.2	166.4 ± 6.9	1.1 (-1.7 to 3.8)	0.442
Weight, kg	63.4 ± 8.8	62.8 ± 9.2	0.6 (-3.1 to 4.3)	0.757
ASA physical status	2 (1-3)	2 (1-3)	0 (0 to 0)	0.866
Surgical site				0.650
Gastrointestinal disease	23 (48.9%)	19 (41.3%)		0.460
Hepatobilitary diseases	18 (38.3%)	22 (47.8%)		0.406
Pancreatic diseases	6 (12.8%)	5 (10.9%)		0.777
Intraoperative data				
Anesthesia time, h	4.3 ± 1.0	4.2 ± 1.0	0.1 (-0.3 to 0.5)	0.773
Sufentanil usage, ug	35.7 ± 6.7	34.6 ± 6.2	1.1 (-1.5 to 3.8)	0.408
Remifentanil usage, ug	1357.4 ± 519.3	1338.2 ± 503.4	19.2 (-191.5 to 229.9)	0.857
Time to extubation, min	10.8 ± 4.4	10.9 ± 4.3	-0.2 (-2.0 to 1.6)	0.852

Notes: Data are number of patients (%), median (range) or median ± standard deviation. Abbreviation: CI, confidence interval.

### Quality of recovery

**Figure 2:**
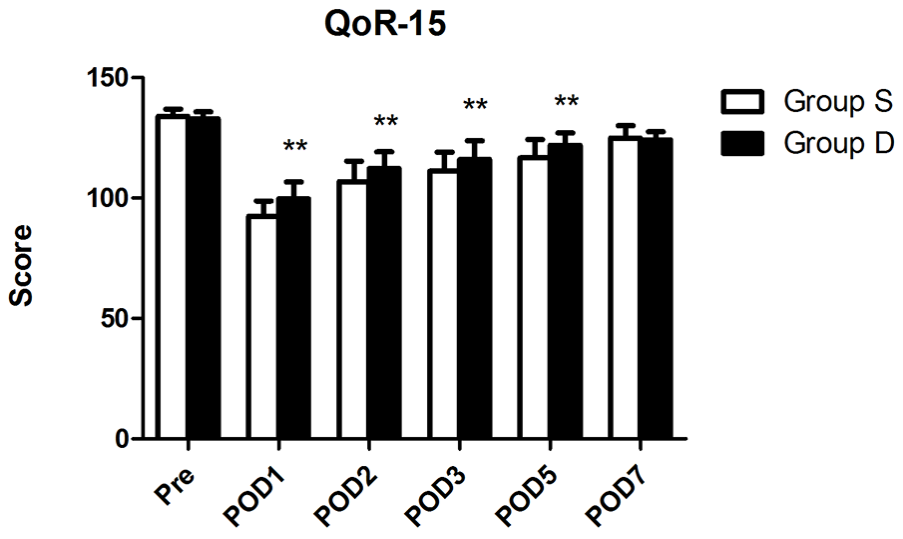
The QoR-15 scores on preoperation, POD1, POD2, POD3, POD5 and POD7 Abbreviation: POD, postoperative day. ^**^ P < 0.01, group S vs. group D.

Baseline QoR-15 scores measured preoperatively did not differ between the two groups (Table 2, Figure 2). The QoR-15 scores were lowest on POD 1 in both groups. The QoR-15 scores were significantly higher in the group D than in the group S on POD 1, 2, 3 and 5 (99.7 ± 6.9 vs 92.5 ± 6.4, 112.3 ± 6.9 vs 106.8 ± 8.5, 116.0 ± 7.8 vs 111.1 ± 8.0, 121.9 ± 5.2 vs 116.7 ± 7.7, respectively. Figure 2), but still lower than their baseline. The dimensions of emotional state, physical comfort and pain were significantly improved in the group D (P < 0.05; Table 2). There is no significant difference between the two groups of psychological support and physical independence.

**Table 2:** Quality of recovery (QoR-15) dimensions and scores

	Group S (n=47)	Group D (n=46)	Difference (95% CI)	P Value
QoR-15 dimensions				
Emotional state				
Preoperative	37.4 ± 1.5	37.2 ± 1.9	0.2 (-0.5 to 0.9)	0.596
POD 1	25.8 ± 3.4	28.8 ± 5.3	-3.0 (-4.9 to -1.2)	0.001^**^
POD 2	34.1 ± 3.0	35.3 ± 1.6	-1.2 (-2.2 to -0.2)	0.018^*^
POD 3	34.4 ± 4.1	36.0 ± 2.5	-1.6 (-3.0 to -0.2)	0.027^*^
POD 5	35.3 ± 2.1	36.7 ± 1.5	-1.4 (-2.2 to -0.6)	0.000^**^
POD 7	36.9 ± 1.0	36.5 ± 1.4	0.4 (-0.1 to 0.9)	0.083
Physical comfort				
Preoperative	41.5 ± 2.2	40.7 ± 2.4	0.8 (-0.1 to 1.8)	0.088
POD 1	35.7 ± 5.6	38.0 ± 4.5	-2.3 (-4.3 to -0.2)	0.034^*^
POD 2	35.8 ± 5.4	38.5 ±5.4	-2.7 (-4.9 to -0.4)	0.019^*^
POD 3	37.2 ± 4.9	39.5 ± 5.0	-2.3 (-4.3 to -0.2)	0.030^*^
POD 5	37.7 ± 6.0	40.3 ± 4.1	-2.6 (-4.8 to -0.5)	0.016^*^
POD 7	41.2 ± 2.4	40.3 ± 2.5	0.9 (-0.1 to 1.9)	0.084
Psychological support				
Preoperative	19.2 ± 0.4	19.1 ± 0.3	0.04 (-0.11 to 0.19)	0.596
POD 1	18.5 ± 0.7	18.6 ± 0.7	-0.1 (-0.4 to 0.2)	0.514
POD 2	18.6 ± 1.9	18.6 ± 2.8	0.1 (-0.9 to 1.1)	0.883
POD 3	18.8 ± 1.4	18.9 ± 0.3	-0.1 (-0.5 to 0.3)	0.692
POD 5	19.2 ± 0.4	19.1 ± 0.4	0.1 (-0.1 to 0.2)	0.468
POD 7	19.1 ± 0.3	19.0 ± 0.1	0.08 (-0.02 to 0.19)	0.098
Physical independence				
preoperative	16.1 ± 0.4	16.3 ± 0.5	-0.18 (-0.36 to 0.01)	0.064
POD 1	1.1 ± 0.2	1.1 ± 0.3	-0.07 (-0.19 to 0.06)	0.284
POD 2	1.4 ± 1.2	1.5 ± 1.5	-0.1 (-0.7 to 0.4)	0.673
POD 3	2.2 ± 2.1	2.5 ± 2.1	-0.2 (-1.1 to 0.6)	0.610
POD 5	5.9 ± 2.3	6.3 ± 1.8	-0.4 (-1.3 to 0.5)	0.345
POD 7	8.0 ± 4.2	8.8 ± 1.6	-0.8 (-2.1 to 0.5)	0.228
Pain				
Preoperative	19.8 ± 0.5	19.6 ± 0.8	0.2 (-0.1 to 0.5)	0.166
POD 1	11.5 ± 2.3	13.2 ± 3.2	-1.8 (-2.9 to -0.6)	0.003^**^
POD 2	16.9 ± 3.1	18.5 ± 2.3	-1.5 (-2.7 to -0.4)	0.007^**^
POD 3	18.5 ± 1.7	19.2 ±1.5	-0.69 (-1.34 to -0.03)	0.041^*^
POD 5	18.6 ± 2.2	19.5 ± 1.1	-0.8 (-1.6 to -0.1)	0.020^*^
POD 7	19.7 ± 0.9	19.6 ± 1.0	0.1 (-0.3 to 0.5)	0.562

Notes: Data are median ± standard deviation. Abbreviation: CI, confidence interval. ^*^ P < 0.05, group S vs. group D, ^**^ P < 0.01, group S vs. group D.

### Postanesthesia care unit information

**Figure 3:**
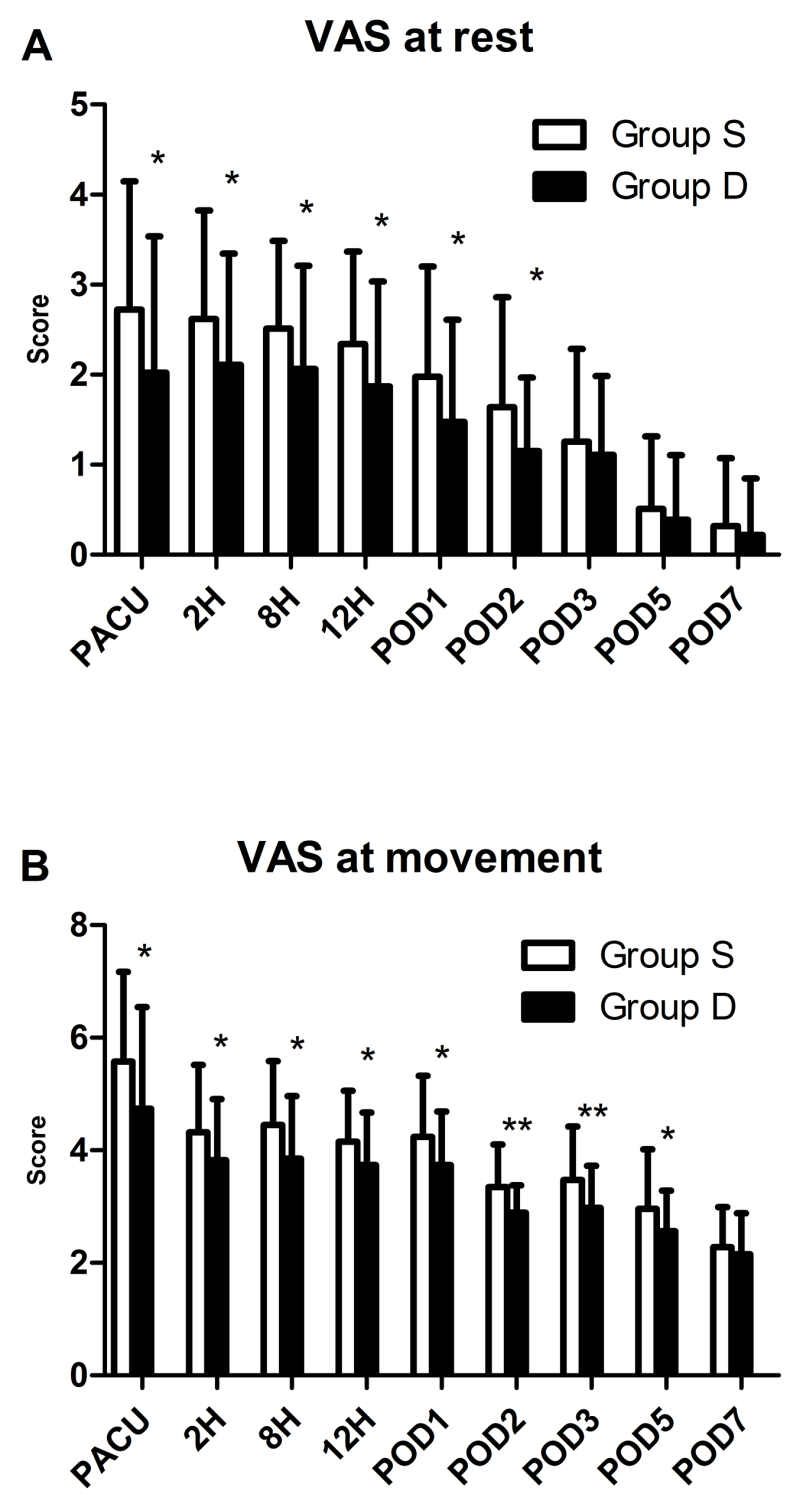
Postoperative pain at rest **(A)**, and at movement **(B)**. Abbreviation: VAS, visual analogue scale (VAS; with 0, no pain, to 10, the worst imaginable pain). ^*^ P<0.05, group S vs. group D, ^**^ P < 0.01, group S vs. group D.

The incidence of nausea in PACU was significantly lower in the group D than in the group S (15.2% vs 36.2%; Table 3). While, the incidence of vomiting and antiemetic drug administered had no difference between the two groups. The VASR was significantly lower in the group D than in the group S (1.4 ± 2.7 vs 1.5 ± 2.0; Figure 3). While, the rescue sufentanil had no significant difference between the two groups (Table 3). The patient satisfaction was significantly higher in the group D than group S (3.2 ± 0.8 vs 2.8 ± 0.8). There was no statistically significant difference between the two groups about the time of discharging from PACU (p > 0.05).

**Table 3:** Postanesthesia care unit parameters

	Group S (n=47)	Group D (n=46)	Difference (95% CI)	P Value
Nausea	17 (36.2%)	7 (15.2%)		0.021^*^
Vomiting	3 (6.4%)	1 (2.2%)		0.617
Administered antiemetic drug	5 (10.6%)	2 (4.3%)		0.435
Administered rescue sufentanil	28 (59.6%)	20 (43.5%)		0.120
Duration in PACU, min	60.6 ± 22.4	67.9 ± 31.6	-7.3 (-18.6 to 4.0)	0.203
Patient satisfaction	2.8 ± 0.8	3.2 ± 0.8	-0.4 (-0.8 to -0.1)	0.012^*^

Notes: Data are number of patients (%) or median ± standard deviation. Abbreviation: CI, confidence interval. ^*^ P < 0.05, group S vs. group D.

### Analgesic effect evaluation

Postoperative pain was assessed with the visual analogue scale (VAS; with 0, no pain, to 10, the worst imaginable pain). The VAS scores were lower in the group D compared with group S within 48 h after surgery (P < 0.05; Figure 3). While, pressing times of analgesic pump and rescue tramadol used had no significant difference between the two groups (P < 0.05; Table 4).

**Table 4:** The PCA button pushed and rescue tramadol required

	Group S (n=47)	Group D (n=46)	P Value
Button pushed on 2h, n (%)			
0/≥1/≥3	25(53.2%)/21(44.7%)/1(2.1%)	26(56.5%)/19(41.3%)/1(2.2%)	0.916
Button pushed on 8h, n (%)			
0/≥1/≥3	24(51.1%)/19(40.4%)/4(8.5%)	24(52.2%)/17(37.0%)/5(10.9%)	0.911
Button pushed on 12h, n (%)			
0/≥1/≥3	30(63.8%)/14(29.8%)/3(6.4%)	33(71.7%)/12(26.1%)/1(2.2%)	0.533
Button pushed on POD 1, n (%)			
0/≥1/≥3	30(63.8%)/13(27.7%)/4(8.5%)	31(67.4%)/10(21.7%)/5(10.9%)	0.808
Button pushed on POD 2, n (%)			
0/≥1/≥3	33(70.2%)/10(21.3%)/4(8.5%)	34(73.9%)/10(21.7%)/2(4.3%)	0.821
Rescue tramadol on 2h, n (%)			
0/≥1/≥2	32(68.1%)/12(25.5%)/3(6.4%)	33(71.7%)/12(26.1%)/1(2.2%)	0.750
Rescue tramadol on 8h, n (%)			
0/≥1/≥2	32(68.1%)/13(27.7%)/2(4.3%)	30(65.2%)/14(30.4%)/2(4.3%)	0.931
Rescue tramadol on 12h, n (%)			
0/≥1/≥2	37(78.7%)/9(19.1%)/1(2.1%)	37(80.4%)/7(15.2%)/2(4.3%)	0.836
Rescue tramadol on POD 1, n (%)			
0/≥1/≥2	38(80.9%)/7(14.9%)/2(4.3%)	33(71.7%)/10(21.7%)/3(6.5%)	0.586
Rescue tramadol on POD 2, n (%)			
0/≥1/≥2	44(93.6%)/3(6.4%)/0(0.0%)	42(91.3%)/3(6.5%)/1(2.2%)	0.837
Rescue tramadol on POD 3, n (%)			
0/≥1	44(93.6%)/3(6.4%)	43(93.5%)/3(6.5%)	1.000
Rescue tramadol on POD 5, n (%)			
0/≥1	44(93.6%)/3(6.4%)	44(95.7%)/2(4.3%)	1.000
Rescue tramadol on POD 7, n (%)			
0/≥1	46(97.9%)/1(2.1%)	44(95.7%)/2(4.3%)	0.617

Notes: Data are number of patients (%).

### Flatus and satisfaction

**Figure 4:**
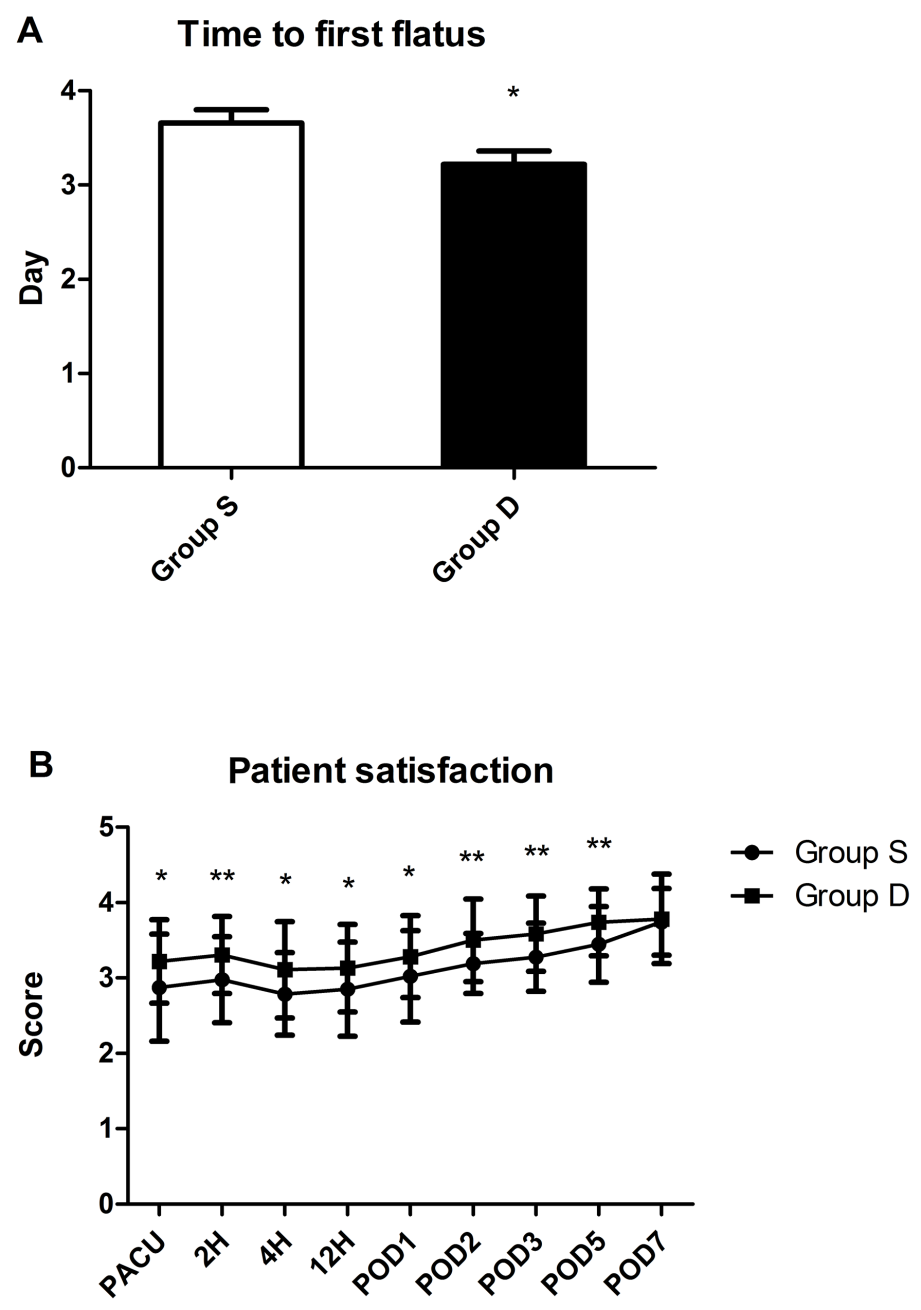
Results of time to first flatus after operation **(A)**, and patient satisfaction **(B)**. Abbreviation: PACU, postanesthesia care unit; POD, postoperative day. ^*^ P<0.05, group S vs. group D, ^**^ P < 0.01, group S vs. group D.

The time to first flatus after surgery was shorter in the group D than in the group S (p < 0.05; Figure 4). The scores of satisfaction of group D were higher than that of group S (p < 0.05).

### Postoperative adverse effects

There were no differences between the two groups in the incidence of postoperative adverse effects with the exception of reduced incidence of nausea within 48 h after surgery in the group D (P < 0.05; Table 5).

**Table 5:** Adverse effects

	Group S (n=47)	Group D (n=46)	P Value
Nausea, n (%)			
0-8H	21 (44.7%)	9 (19.6%)	0.010^*^
0-24H	25 (53.2%)	12 (26.1%)	0.008^**^
0-48H	26 (55.3%)	14 (30.4%)	0.015^*^
Vomiting, n (%)			
0-8H	3 (6.4%)	1 (2.2%)	0.617
0-24H	4 (8.5%)	2 (4.3%)	0.677
0-48H	4 (8.5%)	2 (4.3%)	0.677

Notes: Data are number of patients (%). ^*^ P < 0.05, group S vs. group D, ^**^ P < 0.01, group S vs. group D.

## DISCUSSION

Kehlet is the first one to put forward the concept of Enhanced Recovery After Surgery (ERAS) in 2001 to emphasize earlier recovery after operations [14]. ERAS programs can reduce the rate of surgical complications, reduce hospital costs and increase patient satisfaction [15, 16]. In our study, patient-centered recovery was enhanced significantly with the addition of dexmedetomidine to PCA. Furthermore, dexmedetomidine reduced the incidence of nausea, pain, adverse effect, and improved patient satisfaction after laparotomy surgery.

There is a variety of assessments of QoR in clinical practice, which has become an important outcome of research [17-19]. Patient-centered QoR is superior to other assessments in postoperative period, which can be more intuitively and accurately reflect the patient’s recovery [20]. The QoR-15, patient-centered, evolved from QoR-40, is a 15-item scoring system, an 11-point numerical rating scale (for negative items, 0 = “all of the time” to 10 = “none of the time”; for positive items, the scoring was reversed; score range from 0 to 150), including 5 demensions: physical comfort, physical independence, psychological support, emotional state and pain [12]. QoR-15 can effectively and extensively evaluate postoperative QoR, which can be completed within 3 min and is comparable to the more detailed scale QoR-40 [12, 20].

Dexmedetomidine, as a selective α2-adrenoreceptor agonist, has the characteristics of sedation, anxiolysis, analgesia, and sympatholysis via receptors in locus ceruleus and spinal cord without significant respiratory depression [21, 22]. Studies have reported that dexmedetomidine contributes to early postoperative recovery in the various kinds of surgery including bariatric surgery, thoracic surgery, gynecological laparoscopic surgery, abdominal hysterectomy surgery, abdominal colectomy, thyroidectomy surgery, vertebralsurgery, nasal surgery mastectomy surgery and so on [23-28]. A single-item satisfaction assessment, however, is poorly reliable and is not sufficient to assess postoperative recovery [29, 30]. The QoR-15 scoring system was applied in our study to evaluate the QoR after operation. The scores of QoR-15 were higher in the group D than in the group S. The dimensions of pain, emotional state, and physical comfort were significantly improved in the group D. Furthermore, the scores of patient satisfaction to the early recovery process were significantly higher when dexmedetomidine was used.

The effect of dexmedetomidine on postoperative pain remains controversial. A prospective randomized controlled trial by Cheng et al, evaluated 59 patients who received dexmedetomidine, and reported a significant reduction in pain scores after abdominal operations compared with control group. Other randomized investigations, however, reported no significant difference in postoperative pain [31, 32]. In some clinical studies, intravenous administration of dexmedetomidine presents a postoperative opioid-sparing effect with no reduction in postoperative pain [32]. In the present study, the administration of dexmedetomidine plus sufentanil PCA significantly improved the dimension of pain scores of QoR-15. Furthermore, postoperative VAS pain scores were lower as well. For pressing times of analgesic pump and supplemental requiremanent for tramadol, there were no significant difference between the two groups. However, in the group D, the PCA concentration of sufentanil were half that of the group S. Dexmedetomidine has an anti-nociception effect on skin and visceral pain, which can be reversed by naloxone pretreatment, indicating a possible interaction through opioid systems [33, 34]. A reduction in postoperative PCA opioid requirements may be attributed to the enhanced effect of dexmedetomidine on opioid analgesia [35, 36]. Our results confirmed opioid-sparing effect of dexmedetomidine.

Anxiety is one of the main causes influencing postoperative recovery [37]. In order to enhance recovery and discharge, postoperative physical and psychological stress therapy should be given attention [38]. Studies have indicated that dexmedetomidine provides excellent effect of sedation without respiratory depression [5, 39-41]. In our study, dexmedetomidine beneficially affected the postoperative emotional state (assessed on the QoR-15 dimension of emotion), which was consistent with previously published studies. The improved emotional state in the group D may be induced by the effect of dexmedetomidine on the central nervous system [40]. Dexmedetomidine has the property of anti-inflammatory effect as well, which may contribute to improving emotional state [42, 43]. Furthermore, the analgesic effect of dexmedetomidine can help relieve anxiety as well [5, 44].

The dimension of physical comfort of QoR-15 primarily including nausea and vomiting, sleeping and appetite. In our study, as expected, we observed that the scores in the part of physical comfort of QoR-15 significantly improved in the patients who were administered dexmedetomidine. Previous studies have showed a decrease in the incidence of postoperative nausea and vomiting [45, 46]. In the present study, with consistent to previous clinical trials, the incidence of nausea after operation were reduced in the group D, which may contribute to improve physical comfort of patients. It has been reported that dexmedetomidine has a positive effect on the quality of postoperative sleep without respiratory depression [40, 47]. In our study, the sleep quality was improved in the group D, which could improve the comfortable degree of patients. Surgery has an adverse effect on the movement of the gastrointestinal tract, leading to decreased appetite [48]. In our study, patients administered dexmedetomidine presented a better appetite. Furthermore, the time to first flatus after operation is shorter in the group D, which could promote early recovery of patients.

There are some limitations in our study. First, there is no consensus on the optimal dose of dexmedetomidine contributed to postoperative recovery. The speed of PCA dexmedetomidine at 0.4 ug/(kg·h) is derived from a previous study about abdominal total hysterectomy [46]. Future dose-related studies are needed to establish an optimal dose of dexmedetomidine for early postoperative rehabilitation. Second, all patients in this study used antiemetic drug conventionally, which may affect the antiemetic effect of dexmedetomidine. However, we still observed that the incidence of nausea in the group D was lower than the group S. Third, we did not record the cumulative amount of PCA sufentanil and the rescue tramadol. However, we recorded the frequency of PCA bottom pushed and rescue tramadol used. Finally, studies have demonstrated that dexmedetomidine has a few adverse reactions [31, 49]. In present study, we did not detect a difference in dexmedetomidine-related adverse effects, which is probably related to the low dose of dexmedetomidine. Many clinical researches have showed that small-dose dexmedetomidine infusion resulted in reversible sedation, mild analgesia, reducing the incidence of nausea and vomiting, without inducing adverse effect [50, 51].

In summary, the administration of dexmedetomidine significantly enhanced patient-centered postoperative QoR. The incidence of nausea after operation was reduced, the quality of sleep was improved and that a faster recovery of gastrointestinal function accompanied by a better appetite when dexmedetomidine was administered. Furthermore, the scores of patient satisfaction to the early recovery process were higher with a better control of pain. We recommend the use of dexmedetomidine as an important adjunct to postoperative PCA to improve patient-centered QoR after laparotomy surgery.

## MATERIALS AND METHODS

### Patients and study design

This randomized, double-blind, controlled study was approved by the Ethical Committee of West China Hospital and was registered in the Chinese Clinical Trial Registry (ChiCTR-IPR-16010184). This trial was performed following the Declaration of Helsinki and obtained written informed consent from all subjects before participating in the study.

Patients aged 18-80 years, ASA I to III, scheduled to laparotomy surgery in West China Hospital of Sichuan University, China, between December 2016 and May 2017 were included. Exclusion criteria: (1) patients with atrioventricular block, sinus bradycardia or other serious heart disease; (2) patients with body mass index >30 kg/m2; (3) patients with allergic to the medications used; (4) patients with long history of taking analgesics or antidepressants; (5) patients who had taken other test drugs within three months prior to the study or were involved in other clinical trials; (6) patients who were pregnant or breastfeeding; (7) patients who could not cooperate or refused; (8) patients who were admitted to Intensive Care Unit (ICU) after surgery. Finally, 100 patients were enrolled.

Patients were randomly assigned to the group D (n=50) or group S (n=50) by a computer-generated randomization table. Before the experiment, a total of 100 random numbers were generated according to the 1: 1 ratio of the two groups The grouping was sealed into the sealed envelopes, kept by the operating room pharmacy, which was responsible for the preparation of the study medication. The storage bag of the PCA contained 100 ml solution with the rate of 2 ml/h background infusion, a bolus dose of 0.5 ml and a lock time of 15 min for 48 h after surgery. In the group D, the PCA contained dexmedetomidine and sufentanil, with the infusion rate of 0.04 ug/(kg·h) and 0.02 ug/(kg·h). In the group S, the PCA contained sufentanil only, with the infusion rate of 0.04 ug/(kg·h). The PCA was used to achieve the pain score at rest < 4. All the surgeons, anesthesiologists, nurses, patients and researchers were blinded to the group assignment.

### Anesthetic and surgical management

Before the surgery, patients were informed about the use of the PCA system. Once entering in the operating room, patients were routinely monitored of five-lead electrocardiogram (ECG), pulse oxygen saturation (SPO2), noninvasive blood pressure (BP) and established venous access. Followed by intravenous injection of propofol 2 mg/kg, sufentanil 0.3 ug/kg, and cisatracurium 0.2 mg/kg, endotracheal intubation was performed. Ohmenda-Datex Model 7100 Anesthesia Machine was used for mechanical ventilation (airway peak pressure not more than 40 cmH2O, the oxygen saturation maintained ≥95%, the end-tidal carbon dioxide partial pressure (ETCO2) maintained between 35-45 mmHg). Anesthesia was maintained by inhalation of 1-3% sevoflurane, infusion of remifentanil (0.1-0.2) ug/(kg·min), intermittent administration of cisatracurium and sufentanil to maintain bispectral index (BIS) between 40 and 60. According to the amount of surgical bleeding and blood pressure to adjust the infusion rate and the use of vasoactive drugs, according to the current blood transfusion guidelines to determine the input of blood products. Once started closing the abdominal cavity, PCA system was started and cisatracurium and sufentanil were discontinued. At the same time, ondansctron o.1 mg/kg and tramadol 1.5 mg/kg were administered to prevent postoperative pain, nausea and vomiting. The administration of sevoflurane and remifentanil were discontinued 5 min before the ending of the operation. When the operation was completed, oxygen flow was increased up to 6 L/min in order to quickly wash out sevoflurane. When the patient’s spontaneous breathing tidal volume reached 3 ml/kg, neostigmine 0.04 mg/kg and atropine 0.02 mg/kg were administrated to reverse neuromuscular relaxation. Patients were extubated after recovery from anesthesia, and then transferred to the postanesthesia care unit (PACU).

Once entering the PACU, patients were evaluated the intensity of pain using visual analog scale (VAS), the incidence of bradycardia, shiver, nausea and vomiting every 5 minutes. If the VAS at rest (VASR) ≥ 4, the PCA button was pressed. If the pain still could not be relieved or the VASR ≥ 7, the rescue analgesia of sufentanil 0.1 ug/kg was administrated every 5min until VASR was less than 4. Patients were transferred to ward when Modified Aldrete score ≥ 9.

### Data collection

Our primary outcome was the score of QoR-15 on postoperative day (POD) 3. The QoR-15 questionnaire was conducted on preoperative, POD 1, 2, 3, 5 and 7. The intensity of pain, the rescue analgesics, the incidence of adverse events including bradycardia (HR < 50 beats/min), hypotension (MBP was reduced 20% from the baseline), shiver, nausea and vomiting were evaluated at 2, 8 and 12 h after operation and POD 1, 2, 3, 5 and 7. If the VASR ≥ 4, during 48 h after surgery, the PCA button was pressed. If the pain still could not be relieved, tramadol 100 mg was used intravenous every 30min until VASR was less than 4. If serious adverse events occurred, immediately stop using PCA, and appropriately treated adverse reactions. The time of discharging from PACU, time to first flatus after surgery and the score of patient satisfaction evaluated by a 5-point scale (1, very dissatisfied; 2, not satisfied; 3, neither dissatisfied nor satisfied; 4, satisfied; 5 very satisfied) were also recorded.

### Statistical analysis

The sample size of this study was based on the score of QoR-15 in the 72h after surgery. The score of QoR-15 in the 72h after surgery was 122 (SD 24), which was based on a previous study [13]. 42 patients per group were needed to detect a 10% increase on the score of QoR-15 in the 72h after surgery with an. alpha level of 0.05 and a power of 80%. Considering 20% loss to follow-up rate, 50 patients per group were required. Statistical analyses were performed using SPSS 20.0 (SPSS Inc., Chicago, IL). Continuous data were described as means ± standard deviation. Categorical variables were expressed as percentages. Non-normal distributed data were expressed as median (interquartile range). Continuous data were compared using Student’s t test. Chi-square or Fisher’s exact test was used for categorical data. P value < 0.05 was considered statistically significant.
